# Immuno-oncology: are TAM receptors in glioblastoma friends or foes?

**DOI:** 10.1186/s12964-020-00694-8

**Published:** 2021-01-28

**Authors:** Yunxiang Zhou, Yali Wang, Hailong Chen, Yanyan Xu, Yi Luo, Yongchuan Deng, Jianmin Zhang, Anwen Shao

**Affiliations:** 1 Department of Surgical Oncology, The Second Affiliated Hospital, School of Medicine, Zhejiang University, Hangzhou, 310009 China; 2 School of Pharmacy, Nanjing Medical University, Nanjing, 211126 Jiangsu China; 3 The Second Affiliated Hospital of Zhejiang University School of Medicine (Changxing Branch), Changxing, Huzhou, 313100 Zhejiang China; 4 Department of Neurosurgery, The Second Affiliated Hospital, School of Medicine, Zhejiang University, Hangzhou, 310009 China

**Keywords:** TAM receptors, Glioblastoma, Gas6, Efferocytosis, Immuno-oncology, Cancer immunotherapy, Tumor microenvironment, Janus-faced TAM hypothesis

## Abstract

Tyro3, Axl, and Mertk (TAM) receptors are a subfamily of receptor tyrosine kinases. TAM receptors have been implicated in mediating efferocytosis, regulation of immune cells, secretion of inflammatory factors, and epithelial-to-mesenchymal transition in the tumor microenvironment, thereby serving as a critical player in tumor development and progression. The pro-carcinogenic role of TAM receptors has been widely confirmed, overexpression of TAM receptors is tied to tumor cells growth, metastasis, invasion and treatment resistance. Nonetheless, it is surprising to detect that inhibiting TAM signaling is not all beneficial in the tumor immune microenvironment. The absence of TAM receptors also affects anti-tumor immunity under certain conditions by modulating different immune cells, as the functional diversification of TAM signaling is closely related to tumor immunotherapy. Glioblastoma is the most prevalent and lethal primary brain tumor in adults. Although research regarding the crosstalk between TAM receptors and glioblastoma remains scarce, it appears likely that TAM receptors possess potential anti-tumor effects rather than portraying a total cancer-driving role in the context of glioblastoma. Accordingly, we doubt whether TAM receptors play a double-sided role in glioblastoma, and propose the Janus-faced TAM Hypothesis as a conceptual framework for comprehending the precise underlying mechanisms of TAMs. In this study, we aim to cast a spotlight on the potential multidirectional effects of TAM receptors in glioblastoma and provide a better understanding for TAM receptor-related targeted intervention.

**Video Abstract**

**Video Abstract**

## Introduction

Glioblastoma (GBM) is the most common and fatal primary brain tumor in adults and has a preference for occuring in men and the elderly. GBM accounts for 45.2% of primary malignant brain tumors, with the annual incidence of approximately 3 people per 100,000 person worldwide [1, 2]. GBM develops as a result of a malignant transformation of astrocytoma and represents the most high-grade malignancy of glioma (World Health Organization (WHO) grade IV) [3]. Additionally, GBM is characterized by strong invasiveness, high rates of recurrence and poor sensitivity to therapeutics [4, 5]. In recent decades, continuous advances have been made in the treatment of GBM, including maximal surgical resection, concurrent chemoradiation therapy, adjuvant temozolomide (TMZ) or carmustine wafers, bevacizumab targeted therapy and immunotherapy [6–9]. However, despite the current aggressive treatment protocol, no remarkable improvements have been obtained with regard to the survival rate of GBM patients. Overall, the 2 and 5-year survival rates are still only 27% and 9.8%, respectively, with a mean overall survival of approximately 15 months [9–11].

With newer discoveries and a more in-depth study of cancer immune evasion mechanisms, immunotherapy is appeared to be an effective therapeutic option, in addition to traditional surgery, radiotherapy and chemotherapy [12, 13]. Correspondingly, the successive emergence of immune checkpoint inhibitors (ICIs), such as programmed cell death 1 (PD-1), programmed cell death 1 ligand 1 (PD-L1) and cytotoxic lymphocyte antigen 4 (CTLA4) has achieved a remarkable breakthrough in immuno-oncology [14–16]. Therefore, immunotherapy holds great promise for the treatment of aggressive and malignant GBM, particularly considering that the traditional treatments of GBM are restricted [8, 17, 18]. To date, a myriad of clinical trials concerning GBM immunotherapy have been conducted on a large global scale [19, 20]. Unfortunately, no obvious clinical benefit has been observed thus far [19, 21].

Tyro3, Axl, and Mertk (TAM) receptors are significant players in both the immune and nervous system [22]. Vast literature data indicates the autonomous tumorigenic effect of TAM receptors in tumor immunity microenvironment (TIME), thus TAM inhibition has been explored as a potential anti-tumor strategy a decade ago [23–25]. Similarly, in recent years, the crosstalk between TAM receptors and GBM has increasingly attracted widespread attention. The upregulation of TAM signaling is usually associated with GBM development, progression and poor prognosis [26–28]. Plentiful studies have reached a consensus that TAM receptors have an immunosuppressive and carcinogenic role in the progression of GBM [27, 29–31]. Accordingly, a myriad of clinical trials regarding the specific small molecule inhibitors of Axl in the treatment of recurrent GBM have been registered on *clinicaltrials.gov* and are currently underway, and many combined treatments of anti-TAM therapy and other immunotherapeutic have been carried out [32].

However, most contemporary research focuses on the impact of TAM receptors on tumors, the exploration of changes in tumor immunity remains limited. In this complicated tumor microenvironment (TME), it is almost impossible that one tyrosine receptor kinases (RTKs) family has only one direction of influence on cancer development. Correspondingly, not all evidence supports that blockage of TAM signaling will favor an anti-tumor TME [33]. Especially in some inflammation-driving tumors, TAM blockers may even cause tumor-promoting effects [34]. In addition, it is a surprising finding that TAM signaling serves as adjusters of cancer-related endothelial recruitment, restraining tumor growth through the inhibition of angiogenesis [35]. Importantly, in the context of GBM, the highly malignant and refractory brain tumor that warranting pioneering ideas and span-new treatment strategies, TAM inhibitors have been researched widely. However, several controversial areas remain, including the continued reports that TAM receptor inhibitors have a limited therapeutic effect on GBM and some patients with better prognosis overexpress Mertk receptors [36, 37]. Moreover, recent studies have found that inhibiting inflammation has the potential to substantially prevent the progression of GBM [38]. The emergence of these contradictory observations makes us wonder whether TAM receptors play a dual role in GBM? Therefore, more detailed cellular and molecular mechanisms are urgently needed to further clarify the role of TAM receptors in GBM, so that more precise interventions can be made.

### High heterogeneity and special immune microenvironment of glioblastoma

GBM is the most aggressive and common primary malignant brain tumor in adults [2]. Recent high-throughput data have revealed a wide range of genetic and epigenetic alterations in GBM [39]. According to gene expression profiles, researchers have divided GBMs into multiple different subgroups. Genetic alterations are widespread in GBM, including commonly the loss of heterozygosity (LOH) at 10q, isocitrate dehydrogenase (IDH) mutations, O6-methyl guanine-DNA methyltransferase (MGMT) promoter methylation, epidermal growth factor receptor (*EGFR*) amplification, tumor protein 53 (TP53) mutations [40, 41]. These alterations represent the histological and morphological hallmarks of GBM, encompassing numerous abnormal cell types, increased cell density, local necrosis, and formidable angiogenesis [42]. Gene expression changes and deregulated genetic pathways are also closely related to the biological behavior of the tumor (e.g., rapid proliferation, abnormal differentiation and angiogenesis) and resistance to treatments [43, 44]. These diverse heterogeneity contribute to the difficulty of GBM treatment.

Different from other solid tumors, GBM, which belongs to central nervous system (CNS) tumors, has a unique neuro-immunology known as “immunological privilege” [45, 46]. Besides, the GBM microenvironment lacks lymphocyte infiltration and activated T cells, forming an immunosuppressive TME [46, 47]. Furthermore, the existence of the blood–brain barrier (BBB) prevents chemotherapeutic and immunotherapeutic agents from reaching the tumor site or reaching effective therapeutic concentrations, which is also a potential reason for the failure of some current clinical trials [48, 49]. However, in recent years, with the discovery of the CNS lymphatic vessels [50] and the development of new delivery strategies, e.g., nanoparticle-based drug delivery system [51, 52], for CNS tumor, pharmacotherapy targeting GBM across the BBB seems to be promising [42, 47].

## Overview of TAM receptors

### General features of TAM receptors and their ligands

**Fig. 1:**
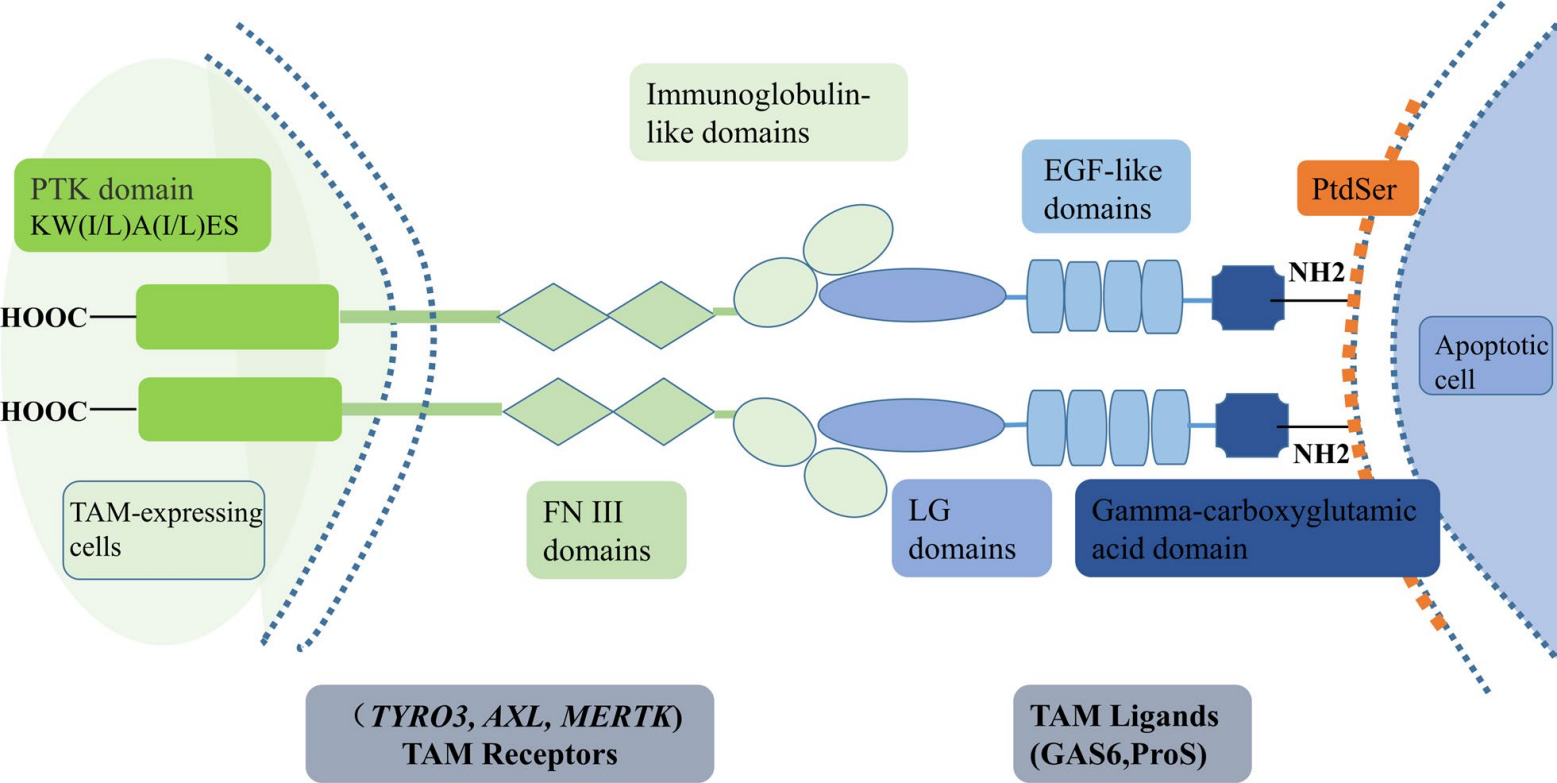
The structural features of TAM receptors and their ligands. TAM receptors have a unique intracellular protein tyrosine kinase domain (named KW(I/L)A(I/L)ES) that distinguishes them from other RTKs. TAM receptors use their extracellular N-terminal immunoglobulin-like domains to bind laminin G (LG) domains of TAM ligands. Gas6 and ProS are the most well-known soluble circulating proteins ligands for TAM receptors, which own a similar N-terminal gamma-carboxyglutamic acid domain structure. TAM receptors, Tyro3, Axl and Mertk receptors; RTKs, receptor tyrosine kinases; Gas6, growth arrest-specific 6; ProS, protein S; FNIII, fibronectin type-III domains. LG domains, laminin G domains; EGF-like domains, epidermal growth factor-like domains

TAM receptors, a subgroup of RTKs family, consist of Tyro3, Axl, and Mertk receptors [53–56]. TAM receptors are distinguished from other RTKs due to the presence of a unique conserved sequence in their kinase domain named KW(I/L)A(I/L)ES and a distinctive extracellular domain, which combines two N-terminal immunoglobulin-like domains followed by two fibronectin type-III (FN-III) domains [53–55]. Therefore, due to the presence of its unique domain, Axl, a 140 kDa protein, was first identified in 1991 [53]. Subsequently, Tyro3 and Mertk were also identified [57, 58]. The two most well-known ligands for TAM receptors include growth arrest-specific 6 (Gas6) and protein S (ProS), which act as bridging factors for TAM receptors indirectly combine with phospholipids including externalized phosphatidylserine (PtdSer, a phospholipid localized in the plasma membrane) on apoptotic cells [59, 60]. The N-terminal gamma-carboxyglutamic acid domain structure of ligands interact with PtdSer,while their laminin G (LG) domains bind to the extracellular immunoglobulin-like domains of TAM receptors, opsonizing downstream TAM signaling functions (Fig. 1) [61, 62]. ProS is only able to bind to Tyro3 and Mertk, Gas6 can bind to all three TAM receptors (Axl > Tyro3 >>> Mertk), whereas, in a specific tumor microenvironment such as the presence of PtdSer, Mertk and Tyro3 are hyperactivated but their affinities for Gas6 are lower than Axl [60, 62, 63].

TAM receptors are widely expressed in human cells, especially in hematopoietic cells, and carry out similar functions [64–67]. They have also been reported to be expressed in the CNS [22, 68, 69], reproductive system [70, 71] and immune system [64, 72]. Interestingly, TAM receptors have been reported to be overexpressed in myeloma cells and acute myeloid leukemia patients as early as two decades ago [73, 74], and have even been shown to participate in disease progression [75].

### The role TAM receptors play in cancer development and immune regulation

Previous studies reveal that TAM receptors regulate the occurrence and development of various diseases [26, 76–78]. There is large quantity evidence showing a relation between autoimmune diseases and abnormal expression of TAM receptors [79, 80]. The absence of the TAM signaling pathway prevents the optimal phagocytosis of apoptotic cells, bringing about disarray in homeostasis that results in autoimmune diseases [79, 81]. The role of TAM signaling has been detected in autoimmune diseases such as rheumatoid arthritis (RA) [82], systemic lupus erythematosus (SLE) [83, 84] and multiple sclerosis (MS) [85]. Furthermore, TAM receptors are also closely associated with several types of human cancer [72]. In particular, high expression of Axl receptor has been observed in advanced colorectal cancer [86], prostate cancer [87] and osteosarcoma [77, 88], which correlates with advanced tumor cell invasion and migration. Moreover, Tyro3 and Mertk receptors have also been found to be upregulated in various tumors such as leukemia and melanoma [74, 89–91]. However, surprisingly, TAM receptors seem to have a two-tier regulatory effect: on the one hand, they promote tumorigenesis and progression; on the other hand, they are implicated in the anti-tumor response of different immune cells [72]. Besides, a report by Wium M et al*.* showed that increased TAM signaling pathway activity was associated with drug resistance, an unfavorable prognosis, and metastasis in cancer patients, while the loss of TAM receptor functions led to the development of autoimmune diseases [26]. Accordingly, TAM receptors play a significant and paradoxical role in oncogenesis and immune regulation.

## The role of TAM receptors in immuno-oncology

### TAM receptors and efferocytosis

TAM receptors are primarily expressed on myeloid hematopoietic cells, including antigen-presenting cells (APCs, such as macrophages and dendritic cells (DCs)), natural killer (NK) cells and platelets [92–94]. In addition to having a significant function in autoimmune disease [80], overexpression of TAMs in various cancers exerts essential roles in macrophage polarization and efferocytosis [25, 95]. Efferocytosis is defined as the process of using phagocytes to accurately recognize and engulf apoptotic cells [96–98]. Apoptosis refers to programmed cell death under physiological or pathological conditions, and phagocytes are capable of recognizing and engulfing apoptotic cells in order to maintain the integrity of the cell membrane and avoid secondary necrosis [96, 99].

**Fig. 2:**
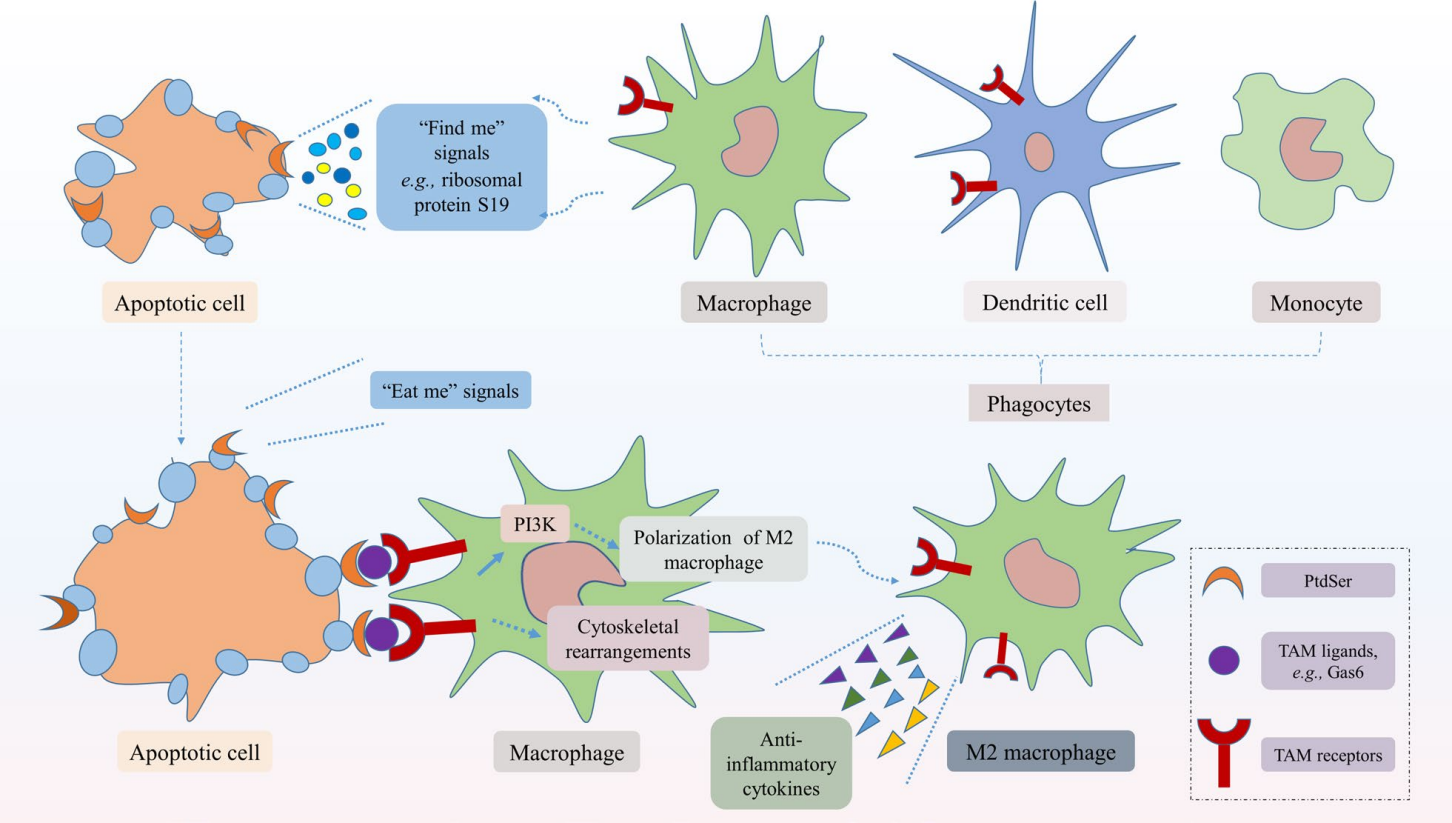
TAM receptor-mediated efferocytosis. Efferocytosis is the process of using phagocytes to accurately recognize and engulf apoptotic cells. Apoptotic cells send out "find me" signals (e.g., lipids, proteins, and peptides) and "eat me" signals (e.g., PtdSer) to promote the recruitment of phagocytes. PtdSer of the apoptotic cells migrates to the outer leaflet of the plasma membrane during apoptosis, interacting with the bridge ligand Gas6/ProS1 to indirectly bind to TAM receptors on the surface of phagocytes. Subsequently, phosphorylation of TAM receptors activates PI3K/Akt and other downstream signaling pathways, regulates cytoskeletal rearrangements to engulf the apoptotic cells, and leads to M2 macrophages polarization, production of anti-inflammatory cytokines. PtdSer, phosphatidylserine; PI3K/Akt, phosphatidylinositol 3 kinase/protein serine threonine kinase; M2 macrophages, M2-like phenotype of macrophages

TAM receptor-mediated efferocytosis was first detected in mice macrophages [71]. Since the overexpression of one or more TAM receptors was identified in various tumor tissues [25], TAM receptor-mediated efferocytosis in the TME has been widely studied, especially Mertk-mediated efferocytosis [100]. Efferocytosis has tumor-promoting functions, including immunosuppression, metastasis and treatment resistance [101]. Efferocytosis is initiated by recognizing apoptotic cells that emit a "find me" signal, thus promoting the aggregation of surrounding phagocytes, including macrophages, monocytes and DCs [102, 103]. Following the “find me” signal, the cell starts to display an "eat me" signal on its surface, urging phagocytes to precisely recognize apoptotic cells [102, 104]. The most extensively studied "eat me" signal is PtdSer. During apoptosis, PtdSer can be transferred from the inner leaflet of the plasma membrane to the outer leaflet [105]. Once there, PtdSer interacts with the bridge ligand Gas6/ProS1, thus indirectly binding to the TAM receptors on the surface of phagocytes (Fig. 2) [62, 106]. As phagocytic receptors, TAM receptors perform different functions, but the activation of Axl and Mertk receptors kinase are indispensably dedicated to the PtdSer-dependent phagocytosis of apoptotic cells [107]. Researchers have found that PS-targeting antibody partially inhibited TAM receptors–mediated efferocytosis [60]. Studies have demonstrated that Axl and Mertk-mediated efferocytosis restrain the innate immune response in macrophages and DCs [103, 108], which creates a TME conducive to tumor development and metastasis [105, 109]. Following the identification and binding of phagocytes to apoptotic cells, TAM receptors become phosphorylated, which leads to the activation of downstream signaling pathways and regulation of cytoskeletal rearrangements, resulting in the engulfment of apoptotic cells [99].

Post-engulfment, under the action of various cytokines, tumor-associated macrophages lean towards M2 macrophage polarization, a wound-healing phenotype, and halting of their anti-tumor immunity [96, 100]. Phosphatidylinositol 3 kinase (PI3K)/protein serine threonine kinase (Akt) pathway is the most common downstream signaling pathway following TAM receptor phosphorylation and contributes to the macrophage polarization [25]. Mechanically, TAM receptors can directly bind to a subunit of PI3K, which causes PI3K to phosphorylates Akt. This leads to macrophage polarization towards an M2 phenotype while dampening polarization of M1 macrophages [110, 111]. On one hand, M2 macrophages encourage the secretion of immunosuppressive cytokines such as transforming growth factor β (TGF-β), interleukin (IL)-10, and IL-13, which recruit regulatory T cells and suppress the response of CD4^+^ and CD8^+^ effector T cells in the TME. On the other hand, they downregulate the expression of pro-inflammatory cytokines such as tumor necrosis factor α (TNF-α), IL-6, IL-12, IL-15, and IL-18 [100, 112]. This change in the expression of cytokines sets up an immunosuppressed TME and promotes tumor progression and invasion, which correlates with poor survival [113].

In addition, the process of efferocytosis can, in turn, upregulates the expression of TAM receptors in tumor APCs, inducing their polarization to immunosuppressive phenotype [100]. The transform in APC phenotypes leads to weakened antigen presentation to T cells, lessened activation of T cells, undermined the effect of antigen-dependent anti-tumor immunity, yielding a more aggressive and tolerogenic TME [100, 114]. Extensive studies have established a consensus that the expression and function of TAM receptors are related to tumor progression, poor survival and drug resistance [22, 23, 25]. Furthermore, Keating AK et al*.* have identified that knockdown of Mertk and Axl receptors enhances the apoptotic response and drug-sensitivity of astrocytoma cells [115]. Overall, the immunosuppression and pro-tumor environment induced by TAM receptor-mediated efferocytosis play an essential role in immuno-oncology.

### TAM receptors regulate PD-L1/PD-L2 expression and are associated with resistance to anti-PD-1 therapy

PD-1 receptor, which is expressed on tumor-infiltrating activated T cells, binds to the ligands PD-L1 and PD-L2 present on APCs. This binding leads to negative regulation of tumor-reactive T cell activation and a weakening of the anti-tumor T cell responses [116–118]. In various kinds of human cancers, it is well-known that PD-1 or PD-L1 and PD-L2 are negative prognostic factors [119–121]. Over the years, in cancer-immunotherapy, studies have found that TAM receptors play key roles in modulating PD-1 axis-related immune checkpoint signals [122].

In 2014, researchers identified that Mertk induces upregulation of PD-L1 transcription in apoptotic cells, which subsequently regulates Mertk-mediated efferocytosis and immune balance for tumor progression [95]. Next, surprisingly, researchers discovered that PtdSer potentiates the effects of PD-L1 signaling to T cells, thus proving the existence of a PtdSer-TAM-PD-L1-PI3k/Akt signaling axis in breast cancer, which contributes to tumor immune escape and chemoresistance [60]. In addition, Mertk significantly upregulated the expression of the coinhibitory ligands PD-L1 and PD-L2 on monocytes/macrophages in the leukemia microenvironment [123]. Inversely, Mertk blockers downregulated the PD-1 receptor on T cells and subsequently induced the activation of tumor-infiltrating T cells, yielding the anti-leukemia immunity [123]. Similarly, Axl was detected to promote epithelial-mesenchymal transition (EMT), which is associated with resistance to anti-PD-1 therapy in metastatic melanoma [124]. A recent analysis has demonstrated that through Axl and PI3K signaling, PD-L1 expression has increased in HPV-negative head and neck squamous cell carcinoma (HNSCC) cells, which correlates with radiotherapy resistance, leading to local treatment failure and higher mortality in HNSCC [125].

Therefore, anti-TAM strategy combined with anti-PD-1/PD-L1 therapeutics represents a novel direction for immune checkpoint inhibitor therapeutics in immune-oncology.

### TAM receptors and associated anti-tumor responses

As mentioned earlier, TAM receptors exert a significant tumorigenic role across a variety of tumors [126]. However, continued studies have revealed that TAM receptor-mediated signaling wields new inhibitory roles in tumor development and angiogenesis [34, 35, 127].

In 2017, Lee EH et al*.* made a surprising discovery that Axl receptors generate effective anti-tumor immunity by upregulating the expression of LIGHT in the T lymphoma TME [127]. LIGHT, a member of TNF superfamily ligand, is a 29 kDa transmembrane protein produced by activated T cells and can compete with herpesvirus envelope glycoprotein D (gD) to bind T-cell herpesvirus entry mediator (HVEM) receptors [128, 129]. LIGHT exerts its immunomodulatory effect by promoting T lymphocyte infiltration, enhancing T-cell proliferation and cytokine secretion and thereby inhibiting tumor growth and progression [130, 131]. In mouse EL4-Axl T lymphoma cells, Axl receptors mediate the expression of LIGHT through the PI3K/Akt/Sp1 axis and promote the secretion of immunocyte regulatory factors such as chemokine C–C motif ligand 5 (CCL5) and its receptor CCR5, thereby enhancing cytotoxic T lymphocytes (CTLs) and NK cells activity in the TME, leading to the suppression of tumor [127].

It is well-known that Axl and Mertk promote the formation of an immunosuppressive microenvironment and tumor evasion immunity by reducing the release of pro-inflammatory factors and inhibiting anti-inflammatory response in the TME [96, 113]. However, evidence also suggests that particular inflammatory conditions can affect tumor promotion [132]. Hence, the definite impact of immuno-inflammatory responses on tumorigenesis remains elusive [133]. Due to its paradoxical nature, researchers have also demonstrated that Axl and Mertk reduce the release of pro-inflammatory factors and limit the phagocytosis of apoptotic neutrophils, thereby inhibiting long-term chronic tumor-promoting inflammation and lowering the incidence of colorectal cancer [34]. Moreover, the inhibitory mechanism of Gas6/TAM in intestinal tumors has also been gradually discovered. Interestingly, one study revealed that Gas6^–/–^ mice possessed stronger azoxymethane/dextran sulfate sodium (DSS)-induced tumorigenesis and poor survival, compared to Gas6^+/+^ mice. The inhibitory effect of Gas6 on intestinal tumors may be related to the suppression of colonic stromal cellular immune response [33]. This study demonstrated that an increase in local Gas6 can activate TLR/Gas6/TAM signaling, limiting the secretion of pro-inflammatory factors such as TNF-α, CXCL1 and CCL2 and the activation of NF-κB. Expression of the pro-tumor factors c-Myc and Cox2 (Ptgs2), which are downstream of NF-κB, were also downregulated. In addition, this pathway also induces the activation of SOCS1/3 and inhibits the immune response of cells that derive from stromal monocyte lineage (such as macrophages) in order to limit intestinal inflammation [33]. In conclusion, Gas6/TAM signaling has been demonstrated to reduce local immune inflammatory responses through the mechanisms outlined above, exert potential intestinal tumor suppression, and prolong the survival of colorectal cancer patients.

In addition, the expression of TAM receptors has also been detected in vascular endothelial cells and vascular smooth muscle cells [82]. Gas6*/*Axl signaling is involved in vascular homeostasis and function via downstream PI3K/Akt signaling [134]. Pros*/*Mertk signaling is engaged in the aggregation, proliferation, migration, invasion of endothelial cell, moreover, TAM signaling inhibits endothelial cell recruitment and angiogenesis, represses vascular endothelial growth factor (VEGF) receptor 2–mediated vascularization [135]. Therefore, TAM signaling exhibits a potential inhibitory role in tumor development through the hindrance of angiogenesis [35, 135].

**Fig. 3:**
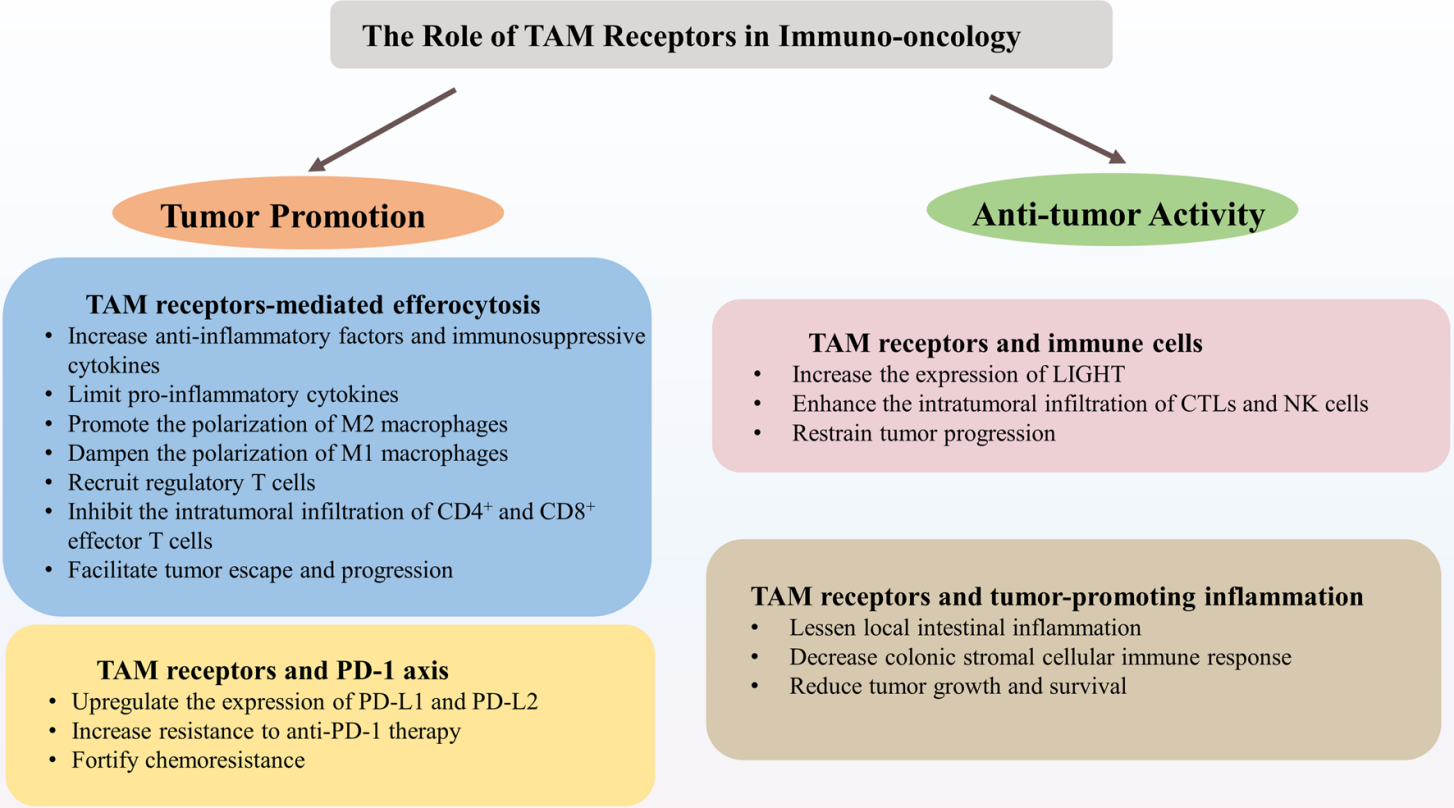
The Role of TAM Receptors in Immuno-oncology. TAM receptors play a bidirectional role in immuno-oncology, not only promoting tumorigenesis but also activating anti-tumor activity. TAM receptors, Tyro3, Axl, and Mertk receptors; TGF-β, transforming growth factor β; IL, interleukin; M2 macrophages, M2-like phenotype of macrophages; PD-L1, programmed cell death 1 ligand 1; PD-L2, programmed cell death 1 ligand 2; CTLs, cytotoxic T lymphocytes; NK cells, nature kill cells

Taken together, TAM receptors may trigger anti-tumor immune responses by activating downstream pathways that enhance the anti-tumor activity of immune cells, lessen local inflammation against inflammation-driving cancers, and impede tumor angiogenesis [35, 135]. Therefore, the overall impact of TAM signaling on carcinogenesis may rest with the combination of all the distinctive cell responses in TME (Fig. 3). In this context, TAM inhibitors may be counterproductive and even promote tumor progression [33].

## Are TAM receptors foes or friends in glioblastoma?

In the past several years, multiple reports have demonstrated the significant roles of TAM receptors in GBM development and prognosis. Thus, they are attractive as innovative therapeutic targets (Table 1) [23, 136, 137]. As limited research concerning TAM receptors in GBM has been conducted, their specific mechanisms of action have not been thoroughly understood yet. Considering the aforementioned contradictory experimental observations concerning TAM receptors in carcinogenesis, we wonder whether TAM receptors are foes or friends in GBM? Is it possible that TAM signaling plays a dual role in GBM?

**Table 1:** Representative agents of TAM-targeted therapy

Agents	Action features	Mechanisms or effects on GBM	References
BGB324	Small molecule inhibitor targeting Axl	Motivate tumor cell apoptosis, suppress GBM proliferation, migration, invasion and survival	[122, 145]
BMS-777607		Increase intratumoral apoptosis, impair neovascularization, proliferation and invasion	[28, 146]
N-butylidenephthalide (BP)	A novel small molecule targeting Axl	Increase gliadel wafer local drug concentration and extend its diffusion distance. Downregulate the expression of Axl and reduce the migratory and invasive capabilities of GBM cells	[137]
TP‐0903	Anti‐Axl antibodies	Intensify sensitivity to TMZ and significantly reverse TMZ resistance in GBM. Promote the proportion of apoptosis and enhance the cytotoxicity of TMZ, thereby dramatically decreasing tumor growth	[144]
AXL-DN	A dominant-negative mutant receptor against Axl	Suppress diffuse-invasive GBM growth and prolong survival	[138]
UNC2025	Orally bioavailable small molecule inhibitor of Mer	Reduce clonal expansion, colony-forming potential, and neurosphere diameter in GBM cells. Possess strong penetration of brain	[136, 140]
Small interfering RNA	Nucleotide aptamer binding to Mer	Cause morphological change of GBM cells, decrease GBM migration and resistance to chemotherapy	[142]
Small interfering RNA	Inducible shRNA-mediated knockdown of Mer and Axl	Increase apoptosis and autophagy, decrease nonadherent colony formation, enhance chemosensitivity	[115]
UNC1062	Pyrazolopyrimidine sulfonamides, small molecule inhibitor against Mer	Inhibit Mer phosphorylation and colony formation, activate anti-tumor immunity	[153]
Foretinib	Multi kinase inhibitor, primarily targeted at c-Met and VEGFR2/KDR; meanwhile, inhibition of Mer and, to a lesser extent, Axl and Tyro3	Inhibit the activation of TAM family receptors and the oncogenic signaling pathways. Decrease cellular survival, migration and invasion of glioma cells	[139]

GBM, glioblastoma; BP, n-butylidenephthalide; TMZ, temozolomide; shRNA, short hairpin RNA; VEGFR, vascular endothelial growth factor; KDR, kinase insert domain receptor; TAM receptors, Tyro3, Axl, Mer receptors

### TAM receptors as foes for glioblastoma patients

Over a decade ago, researchers discovered that TAM receptors and related ligands were highly expressed and activated in GBM tissue, and this was associated with poor prognosis [115, 138]. Nowadays, with the development of clinical utilization of TAM inhibitors, specific blockers of TAM receptors for GBM are gradually entering the field of vision.

In particular, the role of Mertk receptor has been extensively described and has emerged as an attractive therapeutic approach in GBM [139]. Blocking Mertk signals, which creates a pro-inflammatory anti-tumor environment by reducing M2 macrophage polarization, hinders GBM survival and destroys tumor cells [140]. Furthermore, reports have also described that the activation of Mertk plays an important role in GBM growth and invasion, which is the reason why a variety of Mertk inhibitors have been developed to effectively promote cell autophagy and apoptosis and significantly increase the chemosensitivity of GBM to temozolomide [115, 136]. Additional research has demonstrated that overexpression of Mertk receptors in GBM can enhance the infiltration and anti-apoptotic activity of tumor cells [141]. Interestingly, the literature reveals that Mertk signaling mediates the migration of GBM cells and alters cellular morphology, leading to therapeutic resistance in GBM [142].

Similar to Mertk, the role of Axl has also been studied in the context of GBM. Axl and Gas6 are upregulated in gliomas and involved in neovascularization of GBMs, leading to poor prognosis in patients with GBM and reduced recurrence/progression time from 9 to 4 months [143]. Sadahiro H et al*.* first detected that ProS1-mediated Axl signaling, which not only mediates progress and survival of glioma stem cells but also regulates the immune microenvironment, results in aggressive GBM progression [122]. In a GBM model, Wang J et al*.* demonstrated that knockdown of Axl receptor increases TMZ sensitivity and decreases tumorigenesis. Moreover, exogenous Axl upregulation induces TMZ resistance [144]. Therefore, some Axl-targeted inhibitors have been found to effectively block the invasion and migration of GBM [145, 146], and even improve apoptotic response and chemosensitivity [115].

In conclusion, dysfunction of the expression, activation and regulation of TAM family members has been confirmed in GBM. Although the research regarding the specific mechanism of TAM signaling in GBM remains limit, its close relationship with the development, metastasis, prognosis and treatment resistance of GBM has been extensively testified. Thus, To a certain extent, TAM receptors act as foes in the TME of GBM.

### TAM receptors as friends for glioblastoma patients

Interestingly, TAM receptors are not totally harmful to GBM patients. In fact, some studies have gradually manifested the potential inhibitory roles of TAM receptors on GBM progression.

Current research believes that pan-RTK inhibitors (such as Sunitinib), which simultaneously target multiple RTKs, have a better clinical treatment effect in GBM [147, 148]. Surprisingly, a study by Martinho O et al*.* recently identified that activation of Axl by its ligand can modulate the response of sunitinib, causing Axl-positive GBM cell lines to become more sensitive to sunitinib [36]. Therefore, Axl has emerged as having a novel role as a sunitinib response modulator.

Skoda et al*.* analyzed the HGG-02 GBM cell line derived from a patient who experienced a favorable survival outcome and whose event-free survival was nearly 34 months. They observed a significant upregulation of Mertk receptor and down-regulation of Axl phosphorylation in the HGG‑02 cells [37], although a large number of studies have shown that Mertk is correlated with poor prognosis of GBM patients [139]. In brief, this paradoxical results implied that the comprehensive effect of TAM receptors on oncogenesis might rely on the combination of the complicated immune response.

As mentioned earlier, TAM receptors play a tumor-suppressive role in inflammation-mediated tumors [34]. Interestingly, a similar situation may exist in GBM. The notion of immune privilege of the CNS has been reached a consensus for decades. Whereas, in recent years, CNS lymphatics, such as meningeal lymphatic vessels, have been discovered as drainage channels between the CNS and peripheral immune system [50, 149]. Therefore, it provides a possibility for peripheric immune cells, such as T cells and NK cells, to enter into the CNS, which has been demonstrated in the pathological process of meningiomas [150]. Notably, inflammation is an important stimulating factor of GBM [151], and researchers have demonstrated that inhibiting the inflammatory microenvironment in GBM can effectively repress tumor cells proliferation, migration and angiogenesis by activating microRNA-93 [38]. Therefore, TAM receptors, which have been reported to play anti-inflammatory and immunosuppressive roles in the TIME of GBM [122], perhaps have the potential to obstruct the progression of GBM through the regulation of TIME.

On the whole, TAM receptors may be friends in GBM under certain conditions. The positive role of TAM receptors in GBM seems to be confined, on account of numbered studies on the specific mechanism of TAM signaling. However, the contradictory results provide us with innovative ideas of the role of TAM signaling in GBM. As a consequence, the ambivalent role of TAM receptors in GBM needs to be further researched. It is signally important to further clarify the cellular and molecular mechanisms of TAM signaling in GBM.

## Janus-faced TAM hypothesis

Overall, TAM receptors exert multifarious roles in immunity modulation, homeostasis maintenance and tumor progression, in addition to serving as oncogene signals and predictors of poor prognosis in cancer [126, 152]. As is well-known, in various types of tumors, including GBM, the upregulation of TAM signaling is closely linked to tumor invasiveness, metastasis, therapeutic tolerance and poor prognosis [26–28]. Therefore, for treatment of GBM, analogous to other tumors, various TAM inhibitors have been developed and implemented to regulate the immune microenvironment, limit tumor progression, and restore the sensitivity to treatment [115, 142, 153].

In inflammation-driving tumors, *e.g.*, colorectal cancer, it has been shown that the Axl and Mertk receptors have potential cancer suppression effects [64]. They create an inflammation-suppressive immune TME through the TAM signaling pathway, regulate the secretion of immune regulatory factors and activate immune cells, thereby resulting in tumor suppression [34]. In GBM, the mechanism of tumor-promoting inflammation has not yet been detailedly revealed. However, recent studies have shown that inflammation is a driver of GBM, and inhibiting inflammation can effectively curb tumor invasion [38, 151]. Therefore, in the context of GBM, the immunosuppressive and anti-inflammatory TAM receptors may also portray an anti-tumor role under certain circumstances. Besides, TAM signaling has potential anti-tumor effects by suppressing angiogenesis [35], a necessary condition for tumor nutrition, metabolism and metastasis, although this anti-angiogenic efficacy has not been verified in GBM.

As is mentioned above, it is interesting that researchers detected that Axl is a modulator in GBM, as it regulates the therapeutic sensitivity of GBM cells to sunitinib [36]. Researchers have also detected that the expression of Mertk is upregulated in a GBM patient with a good prognosis [37]. Importantly, Tyro3 is relatively highly expressed in CNS compared with Axl and Mertk [27], however, there are less pointed studies. Hence, some potential regulative mechanisms may not have been discovered yet in TIME.

Overall, in immuno-oncology, TAM receptors have received extensive attention. However, their specific mechanisms and predictive biomarkers of efficacy remain to be fully elucidated. In this study, we discuss the potential dual effect of TAM receptors and put forward a Janus-faced TAM Hypothesis to understand the potential two-tier role of TAM receptors in GBM, thus providing a fresh perspective for the treatment of this aggressive tumor. On one hand, they motivate GBM immune escape and resistance to therapy. On the other hand, they have the potential to activate GBM-related immune cells and inhibit tumor angiogenesis, thus yielding anti-tumor effects and prolonging survival.

## Conclusion and perspectives

TAM receptors are widely expressed in human cells and are upregulated in various tumors. They can indirectly bind to PtdSer through bridging ligand to mediate efferocytosis, which induces an immunosuppressive environment for tumor survival and growth. Moreover, TAM receptors upregulate the expression of PD-L1 and PD-L2 and increase resistance to anti-PD-1 therapy. However, they can also portray anti-tumor roles by modulating the activity of immune cells and inhibiting angiogenesis. Similarly, in the context of GBM, TAM receptors seem to be a key player in tumor cell growth, metastasis, invasion, and treatment-resistance. Nevertheless, conflicting observations and complicated TME imply that TAM receptors may not only play a one-way cancer-promoting effect in GBM. They positively modulate the therapeutic sensitivity of pan-RTK inhibitors. More importantly, they impede tumor angiogenesis and even also induce anti-tumor immune response under certain conditions. Accordingly, we first propose the Janus-faced TAM Hypothesis to uncover the potential bidirectional role of TAM receptors in GBM and provide a new research direction for this highly malignant and refractory glioma.

TAM-dependent immunomodulatory functions are attractive strategies for cancer immunotherapy. Nevertheless, the different regulatory roles of TAM receptors are dependent on the intricate cellular context. Tumor status, level of inflammation, and the type of immune cells in TME may possess a paradoxical dual role during the treatment of different tumors. Actually, most clinical trials regarding TAM receptors target Axl. In view of the context-dependent characteristics of TAM and the unique molecular signaling mechanism of the three receptors, targeting one or a combination of multiple TAM receptors may have different therapeutic effects, which indeed warrants necessary further research in the future. Hence, due to the duality of TAM receptors, future studies will have to focus on how to determine the sensitivity of selected patients, the efficacy-associated predictive biomarkers, and how to implement precise treatment. Besides, although it has been reported that TAM receptors can be revitalized by a virus infection, and TAM agonists play potential roles in preventing viral encephalitis [134], how to specifically activate TAM receptors is still a challenge.

Furthermore, TAM receptors are involved in the PD-1 axis-related therapeutic resistance and regulate diverse immune cells to exert anti-tumor immunity in selected tumors. Yet, these mechanisms have not been discovered in GBM. Consequently, further research is needed. Additionally, the highly intratumoral heterogeneity lead GBM insensitive to single-targeted therapy or single-agent therapy, so appropriate drugs combination with TAM-targeted therapy is worth exploring. Therefore, combined treatment with another immunotherapy such as anti-PD-L1 treatment appears promising for TAM-based cancer immunotherapy.

## Data Availability

Not applicable.

## Abbreviations

TAM: Tyro3, Axl, and Mertk receptors
GBM: Glioblastoma
WHO: World Health Organization
TMZ: Temozolomide
ICIs: Immune checkpoint inhibitors
PD-1: Programmed cell death 1
PD-L1: Programmed cell death 1 ligand 1
CTLA4: Cytotoxic lymphocyte antigen 4
TIME: Tumor immunity microenvironment
TME: Tumor microenvironment
RTKs: Receptor tyrosine kinases
LOH: Loss of heterozygosity
IDH: Isocitrate dehydrogenase
MGMT: O6-methyl guanine-DNA methyltransferase
EGFR: Epidermal growth factor receptor
TP53: Tumor protein 53
CNS: Central nervous system
BBB: Blood–brain barrier
FN III domains: Fibronectin type-III domains
Gas6: Growth arrest-specific 6
ProS: Protein S
PtdSer: Phosphatidylserine
LG domains: Laminin G (LG) domains
RA: Rheumatoid arthritis
SLE: Systemic lupus erythematosus
MS: Multiple sclerosis
APCs: Antigen presenting cells
DCs: Dendritic cells
NK cells: Natural killer cells
M2 macrophages: M2-like phenotype of macrophages
PI3K/Akt pathway: Phosphatidylinositol 3 kinase/protein serine threonine kinase pathway
M1 macrophages: M1-like phenotype of macrophages
TGF-β: Transforming growth factor β
IL: Interleukin
TNF-α: Tumor necrosis factor α
EMT: Epithelial-mesenchymal transition
HPV: Human papillomavirus
HNSCC: Head and neck squamous cell carcinoma
gD: Herpesvirus envelope glycoprotein D
HVEM: Herpesvirus entry mediator
CCL5: Chemokine C–C motif ligand 5
CTLs: Cytotoxic T lymphocytes
DSS: Dextran sulfate sodium
VEGF: Vascular endothelial growth factor
